# Momordica charantia (bitter melon) attenuates high-fat diet-associated oxidative stress and neuroinflammation

**DOI:** 10.1186/1742-2094-8-64

**Published:** 2011-06-03

**Authors:** Pratibha V Nerurkar, Lisa M Johns, Lance M Buesa, Gideon Kipyakwai, Esther Volper, Ryuei Sato, Pranjal Shah, Domonkos Feher, Philip G Williams, Vivek R Nerurkar

**Affiliations:** 1 Laboratory of Metabolic Disorders and Alternative Medicine, Department of Molecular Biosciences and Bioengineering, College of Tropical Agriculture and Human Resources, University of Hawaii at Manoa, Honolulu, HI, 96822, USA; 2 Retrovirology Research Laboratory, Department of Tropical Medicine, Medical Microbiology and Pharmacology, John A. Burns School of Medicine, University of Hawaii at Manoa, Honolulu, HI, 96813, USA; 3 Department of Chemistry, College of Natural Sciences, University of Hawaii at Manoa, Honolulu, HI, 96822, USA

## Abstract

**Background:**

The rising epidemic of obesity is associated with cognitive decline and is considered as one of the major risk factors for neurodegenerative diseases. Neuroinflammation is a critical component in the progression of several neurological and neurodegenerative diseases. Increased metabolic flux to the brain during overnutrition and obesity can orchestrate stress response, blood-brain barrier (BBB) disruption, recruitment of inflammatory immune cells from peripheral blood and microglial cells activation leading to neuroinflammation. The lack of an effective treatment for obesity-associated brain dysfunction may have far-reaching public health ramifications, urgently necessitating the identification of appropriate preventive and therapeutic strategies. The objective of our study was to investigate the neuroprotective effects of *Momordica charantia *(bitter melon) on high-fat diet (HFD)-associated BBB disruption, stress and neuroinflammatory cytokines.

**Methods:**

C57BL/6 female mice were fed HFD with and without bitter melon (BM) for 16 weeks. BBB disruption was analyzed using Evans blue dye. Phosphate-buffered saline (PBS) perfused brains were analyzed for neuroinflammatory markers such as interleukin-22 (IL-22), IL-17R, IL-16, NF-κB1, and glial cells activation markers such as Iba1, CD11b, GFAP and S100β. Additionally, antioxidant enzymes, ER-stress proteins, and stress-resistant transcription factors, sirtuin 1 (Sirt1) and forkhead box class O transcription factor (FoxO) were analyzed using microarray, quantitative real-time RT-PCR, western immunoblotting and enzymatic assays. Systemic inflammation was analyzed using cytokine antibody array.

**Results:**

BM ameliorated HFD-associated changes in BBB permeability as evident by reduced leakage of Evans blue dye. HFD-induced glial cells activation and expression of neuroinflammatory markers such as NF-κB1, IL-16, IL-22 as well as IL-17R were normalized in the brains of mice supplemented with BM. Similarly, HFD-induced brain oxidative stress was significantly reduced by BM supplementation with a concomitant reduction in FoxO, normalization of Sirt1 protein expression and up-regulation of Sirt3 mRNA expression. Furthermore, plasma antioxidant enzymes and pro-inflammatory cytokines were also normalized in mice fed HFD with BM as compared to HFD-fed mice.

**Conclusions:**

Functional foods such as BM offer a unique therapeutic strategy to improve obesity-associated peripheral inflammation and neuroinflammation.

## Background

The rising epidemic of obesity is associated not only with diabetes and cardiovascular diseases, but also brain dysfunction and early onset of dementia [1]. High-fat diet (HFD) induces pro-inflammatory cytokines including tumor necrosis factor α (TNFα), interleukin (IL-1β) and IL6 in peripheral tissues as well as hypothalamus, raising the possibility of a cross-talk between peripheral and neuroinflammation [2,3]. Negative impact of increased body mass on brain and its function is evident from impaired cognition [4], a lower brain volume [5], increased lesions of the white matter [6], temporal lobe atrophy [7], and Alzheimer-type neuropathological changes [8] among obese individuals. In rodents, diet-induced obesity compromises spatial learning skill [9], reduces hippocampus plasticity [10], and contributes to the development of Alzheimer's disease (AD) [11]. Although the underlying mechanisms remain unclear, stress and neuroinflammation have been implicated to play a major role in diet-induced brain dysfunction [12,13].

Regardless of our present knowledge involving cellular pathways that modulate obesity-associated neurodegenerative diseases, complete therapeutic prevention or reversal of acute or chronic neuronal injury remains elusive. Therapeutic and preventive strategies to reduce obesity require multiple therapies and life-style changes that may not necessarily ameliorate neuronal insults. Interestingly, Okinawan centenarians demonstrate normal cognitive function such as attention span and memory, and delayed onset of dementia, which was associated with various life-style factors including consumption of bitter functional foods [14,15].

Our study investigates the neuroprotective effects of *Momordica charantia *(bitter melon, BM), a staple of traditional Okinawan diet, to ameliorate obesity-associated neuroinflammation and stress. BM is widely cultivated in Asia, Africa and South America and extensively used in Ayurvedic and Chinese medicines as a remedy for diabetes and its complications including neuropathy [16]. We and others have demonstrated that BM reduces adiposity in rodents fed a HFD, lowers plasma and hepatic lipids, insulin and leptin levels and normalizes glucose tolerance [17-19]. Furthermore, BM has been demonstrated to lower systemic oxidative stress in streptozotocin-induced diabetic rats [20] as well as proinflammatory interleukins in lipopolysaccharide (LPS)-stimulated murine peritoneal macrophages [21]. We therefore tested the hypothesis that BM will lower HFD-associated systemic inflammation as well as neuroinflammation and oxidative stress.

Our data suggests that BM attenuated HFD-induced BBB disruption and mRNA expression of neuroinflammatory cytokines such as IL22, IL16 and NF-κB1 with a concomitant reduction in oxidative stress in brains of mice fed HFD for 16 weeks. Amelioration of HFD-associated neuroinflammation by BM was accompanied by increased expression of sirtuin 1 (Sirt1) transcripts and reduction of forkhead box class O transcription factor (FoxO) mRNA, known to regulate oxidative stress and inflammation. Furthermore, BM attenuated plasma oxidative stress markers and peripheral inflammation, specifically modulating Th1 vs Th2 immune response and possibly activating Th17 lymphocytes. BM also reduced circulating plasma cytokine levels including monocyte chemoattractant protein 1 (MCP1), macrophage colony-stimulating factor (M-CSF), and macrophage inflammatory protein-1α (MIP-1α) that are involved in either disrupting the blood-brain barrier (BBB) or inducing chemotaxis of T lymphocytes and monocytes in vascular endothelial cells of the BBB [22-24]. Overall, functional foods such as BM offer unique possibilities to ameliorate not only obesity and type 2 diabetes (T2D), but also may simultaneously prevent and/or delay onset of obesity-associated systemic inflammation, neuroinflammation and stress.

## Methods

### Preparation of BM

Details of BM preparations have been described previously [19]. In brief, Chinese variety of young BM (raw and green) was obtained from local farmer's market, juiced using a household juicer, freeze dried at -45°C for 72 h and stored at -80°C until used for feeding studies. The Chinese BM was verified and identified by ethnobotanist, Dr. Will McClatchy (Professor, Dept. of Botany, University of Hawaii) and voucher specimens were deposited at official herbaria, University of Hawai'i at Manoa, herbarium (HAW), and labeled as PratibhaBM0001 and PratibhaBM0002.

### Animal studies

All animal experiments and procedures were conducted in accordance with guidelines established by the National Institutes of Health and the University of Hawaii Institutional Animal Care and Use Committee. Four- to six -weeks old female C57BL/6 mice were purchased from Jackson Laboratories (Ann Harbor, MI) and randomized in four groups of eight animals each: 1) control, 2) control diet + 1.5% freeze dried BM (w/w), 3) HFD, and 4) HFD + 1.5% freeze dried BM (w/w). Brains from four animals of each group were used for biochemical analysis. Details of animal experiment and diets have been described previously [19]. In a separate set of experiments, four animals were used to analyze the BBB permeability. As published earlier, control rodent chow contained 11 kcal% fat (4.07 kcal/g, #D12328, Research Diets, New Brunswick, NJ), whereas HFD chow contained 58 kcal% fat (5.6 kcal/g, # D12331, Research Diets) [19]. Freeze dried BM juice was mixed with diet at 1.5%, w/w. Macronutrient analysis demonstrated that lyophilized BM extract contained 24% protein and 2% fat [19]. However, changes in protein, carbohydrate or fat content of the diet were negligible with addition of 1.5% (w/w) BM. Therefore the composition of control or HFD diet was not modified. Addition of BM did not significantly change either the carbohydrate or mineral content of the diet, based on the macronutrient chemical analysis data published earlier [19]. Food and water was provided *ad libitum*. Food intake and body weights were measured daily while water intake was measured weekly as described previously [19]. After 16 weeks, mice were anesthetized using 2% Attane™ isoflurane (Minrad Inc, Bethlehem, PA) under 2 LPM oxygen, using OHMEDA Isotec 4 Vaporizer (Datex-Ohmeda, Helsinki, Finland). Animals were perfused with ice-cold phosphate-buffered saline (PBS) to flush off blood from the brain and other organs. Whole brains were isolated after euthanasia, washed in PBS and immediately snap frozen in liquid nitrogen and were stored at -80°C until further analysis.

### Determination of BBB permeability

Influence of HFD and BM on BBB permeability was determined by measuring Evans blue dye transport across the BBB according to published protocols [25,26]. In brief, Evans blue dye (50 mg/kg) was injected intraperitoneally. After 3 h, mice were anesthetized and perfused with PBS. Brains were quickly dissected and snap frozen in liquid nitrogen. Right brain was crushed on dry ice and the powder was homogenized with Bullet Blender (Next Advance, Inc., Averill Park, NY) in one mL of 50% trichloroacetic acid (TCA, wt/vol) using 0.5 mm stainless steel beads (Next Advance, Inc.) for 90 seconds. Homogenates were centrifuged at 10,000 rpm for 20 minutes. The supernatant was diluted with 100% ethanol (1:2) and 200 μl was added to 96-well black plates. Fluorescence was monitored using Wallac Victor^2 ^1420 Multilabel Counter (PerkinElmer Life Sciences, Boston, MA) at 620-nm excitation and 680-nm emission. Total dye was calculated based on external standards (10 -1000 ng/mL) dissolved in 50% TCA/ethanol (1:3).

### RNA extractions

Frozen left brains were powdered to obtain a homogenous sampling and 30 to 40 mg of whole brain was homogenized using PRO 200 Laboratory Homogenizer (Pro Scientific, Oxford, CT) followed by the QIAshredder (Qiagen, Inc., Valencia, CA, Cat#: 79656). RNA was extracted using the RNeasy Mini Kit (Qiagen, Inc., Cat#: 74104) as per the manufacturers' protocol. The quality of RNA was tested using Agilent bioanalyzer at the Greenwood Molecular Biology Facility, Pacific Biosciences Research Center, University of Hawaii at Manoa (UHM). The 18S/28S ratios of all samples were in the range of 1.8 to 2.0.

### Genome-wide microarray analysis

Transcriptional profiling of mouse brain was conducted using Affymetrix GeneChip Mouse Genome 430 2.0 Array (Affymetrix, Inc., Santa Clara, CA. Part#: 900497) according to the standard GeneChip eukaryotic target labeling protocol (Affymetrix, Inc.), at the Greenwood Molecular Biology Facility, UHM. All subsequent manipulations, including labeling and hybridization, were performed independently for each sample. Briefly, 10 μg of total cellular RNA per sample was used to synthesize double-stranded cDNA, which then was transcribed *in vitro *in the presence of biotinylated dNTPs (Enzo Diagnostics, Farmingdale, NY). Successful labeling of all the samples was tested by test array hybridizations, using the Affymetrix GeneChip Test3 Array (Affymetrix, Inc., Part#: 900341), to ensure quality of the biotinylated target. Appropriate internal controls were added to the hybridization cocktail. Gene chip arrays were scanned at 570 nm with a gene array scanner and analyzed with Gene Chip Operating Software (GCOS) v1.4 (Affymetrix, Inc.). The method of normalization used was a scaling algorithm, which involves multiplying the mean intensity of each chip (not including the upper and lower 2%) by a factor, which changes the mean intensity to 500 for every chip. By scaling each chip, a direct comparison could be made between all the chips. For a given gene transcript in any chip-to-chip comparison GCOS generates a *Change Call *parameter *Increase *or *Decrease *based on a consideration of signal specificity as well as intensity. The *Change Call *is based on an evaluation of the intensities of the signals generated from each gene transcript on one chip relative to the corresponding signal intensities on the other chip. The data were filtered using Genespring 9.0 software, based on both "Detection Call" (presence or absence) and "Signal Log Ratio" (1.5-fold change or greater) in any of the comparisons between control and various treatment groups. In the case of the Affymetrix Detection Call algorithm, genes that were "*Absent" *in all samples were filtered out. Genes had to be detected or "*Present" *in at least one out of four treatment groups.

### Validation of selected genes by real-time RT-PCR

To confirm changes in the expression of selected genes, RNA samples from four mice in each treatment group, including those analyzed by microarray, were assayed by quantitative real-time reverse transcriptase-PCR (qRT-PCR) using SYBR Green (Bio-Rad iCycler, Hercules, CA). In brief, one μg of total RNA was used for cDNA synthesis (iScript cDNA Synthesis Kit, Bio-Rad, Hercules, CA) as described previously [27]. qRT-PCR was conducted by using two μL of diluted cDNA (1: 3) in duplicate with both internal and no template controls. Primer sequences used for amplification of specific genes were designed using the Beacon Designer 2.0 primer design software (PREMIER Biosoft International, Palo Alto, CA, Table 1). A SYBR Green detection system was used and the reactions generated a melting temperature dissociation curve enabling quantitation of the PCR products. The housekeeping gene GAPDH, whose expression was unaffected by HFD, was used as an internal control for normalization in parallel with each gene of interest. PCR cycling conditions were 95°C for 5 min, 45 cycles of 95°C for 10 sec, 54°C for 45 sec except for Eif3s10 (95°C for 5 min, 45 cycles of 95°C for 10 sec, 56°C for 45 sec), and the melt curve beginning at 55°C of 80 cycles and gradually increasing 0.5°C every 10 sec. To verify reproducibility, each brain sample was analyzed in duplicate in two independent experiments for each gene. PCR product intensity data were normalized to GAPDH and relative fold-change was obtained using the ΔΔCt equation calculated by the Bio-Rad iQ5 software.

**Table 1:** Primer sequences for real-time RT-PCR

Target Gene (GenBank Acc. No.)	Primer Sequence	Amplicon size (bp)
**IL-22 **(AJ249492) *Forward* *Reverse*	5'-GTGACGACCAGAACATCCAG-3' 5'-ATCTCTCCGCTCTCTCCAAG-3'	76

**IL-17rb **(NM_019583) *Forward* *Reverse*	5'-CTGTGTGGAGGTAGTGCTATG-3' 5'-CTCAGGCGTGTATGCTCAG-3'	78

**NF-κB **(AK011965) *Forward* *Reverse*	5'-CGTCGGTGCTATTCTGTTG-3' 5'-GCTGTCTGGTAAAGGTTGTTC-3'	76

**IL-16 **(BC026894) *Forward* *Reverse*	5'-AGGAAAGCAAGATGGAGGAC-3' 5'-GAGGAGTTAGGGAAGGTTGTG-3'	116

**Aif1/Iba1 **(NM_019467.2) *Forward* *Reverse*	5'-GTCCTTGAAGCGAATGCTGG-3' 5'-CATTCTCAAGATGGCAGATC-3'	157

**CD11b **(NM_019467) *Forward* *Reverse*	5'- CAGGCATCACTTCCACATCAGC-3' 5'- CTGTCTTAACCTGCATCA-3'	174

**GFAP **(NM_10277) *Forward* *Reverse*	5'- TCTCCTCCTCCAGCGATTCAAC-3' 5'- TGTGGATTTGGAGAGAAA-3'	78

**S100B **(NM_009115) *Forward* *Reverse*	5'- CTGCTTGCCATCTGCCTGAG-3' 5'- GAGGTTGCTCATCCTTAC-3'	134

**Eif3s10 **(AW701127) *Forward* *Reverse*	5'-CTCTTCAGTCCGGTCATCTTTC-3' 5'-CAAGTCGCCGTGATGATAGG-3'	194

**Hsp110 **(D67017) *Forward* *Reverse*	5'-AATGAGAAGGGCTCTGTCAAC-3' 5'-CAGGATCACATACTATGGCAAAC-3'	96

**Dnajb1 **(AK002290) *Forward* *Reverse*	5'-AGTGGTGGTGCTAATGGTAC-3' 5'-AAAAGGTATCAAAGGGGTTTCTG-3'	103

**Dnaja3 **(AK004575) *Forward* *Reverse*	5'-AGATGGTCAGACTGTAAGGATG-3' 5'-AGAGGTCCGAGTGGATGTC-3'	113

**Sirt3 **(NM_022433) *Forward* *Reverse*	5'-CTGGATGGACAGGACAGATAAG-3' 5'-TCTTGCTGGACATAGGATGATC-3'	79

**Foxo1 **(AI462296) *Forward* *Reverse*	5'-ACGGGCTGTCTGTCTGTC-3' 5'-TAAGTGAAGTTTCTCTGTGGTTTC-3'	116

**Foxo3a **(BB364488) *Forward* *Reverse*	5'-GCCTCGGTCACACTCCAG-3' 5'-TTCACACCTGTCCACATTTCC-3'	166

**GAPDH **(NM_001001303) *Forward* *Reverse*	5'-TCAACGGCACAGTCAAGG-3' 5'-ACTCCACGACATACTCAG-3'	126

### Brain superoxide dismutase activity

Brain homogenates were prepared in 10 volumes of ice-cold HEPES buffer (20 mM HEPES, 1 mM EGTA, 210 mM mannitol, 70 mM sucrose, pH 7.2) and centrifuged at 1,500 × g for 5 min. The supernatant was further centrifuged at 10,000 X g for 15 min. The resulting supernatant contained the cytosolic superoxide dismutase (SOD), and the pellet containing the mitochondrial SOD was resuspended in HEPES buffer. Both fractions were frozen at -80°C for up to one month, and cytosolic (Cu/ZnSOD) and mitochondrial SOD (MnSOD) activities were analyzed using commercial Superoxide Dismutase Assay kit (Cayman Chemicals, Ann Arbor, MI).

### Brain catalase activity

Brain homogenates (20% w/v) were prepared in ice-cold phosphate buffer containing 50 mM potassium phosphate, pH 7.0 containing 1 mM EDTA and centrifuged at 10,000 X g for 15 min at 4°C. The supernatants were collected and stored at -80°C until analysis for up to one month. Catalase activity was assayed using commercial Catalase kit according to manufacturers' protocol (Cayman Chemicals).

### Brain glutathione peroxidase (GPx) activity

A 10% brain homogenate was prepared in ice-cold buffer containing 50 mM Tris-HCl, pH 7.6, containing 5 mM EDTA and 1 mg/mL BSA and centrifuged at 10,000 X g for 15 min at 4°C. Supernatants were collected and frozen at -80°C until analysis for up to one month. GPx activity was measured using commercial GPx kit according to the manufacturers' protocol (Cayman Chemicals).

### Brain GSH levels

Total brain GSH was measured according to published protocol [28] with modifications [29]. In brief, a 20% w/v homogenate was prepared from frozen brain using phosphate buffer containing 100 mM sodium phosphate, pH 8.3, and 5 mM EDTA. Homogenates were centrifuged at 10,000 X g for 15 min and supernatants were deproteinated with an equal volume of 10% trichloroacetic acid and frozen at -80°C until analysis. The final reactions were performed in a 96-well plate by incubating deproteinated brain homogenates, phosphate buffer and o-phthalaldehyde solution in a total volume of 200 μL. Fluorescence was monitored at 350/420 in the Wallac Victor^2 ^1420 Multilabel Counter (PerkinElmer Life Sciences) and values were compared to GSH standard curve freshly prepared with each batch of samples and adjusted to μM GSH/g brain.

### Analysis of glial cell activation

Ionized calcium-binding adapter molecule 1 (Iba1) and integrin αM (CD11b) mRNA expression, were measured to analyze microglial cells activation, whereas glial fibrillary acidic protein (GFAP) and calcium binding protein, beta (S100β), mRNA expression were measured to quantitate astrocyte activation. mRNA was analyzed by qRT-PCR as mentioned above, using primers and conditions described in Table 1.

Iba1 and GFAP protein levels were analyzed by western blotting. Whole cell proteins were extracted by preparing a 5% homogenate in ice-cold buffer containing 10 mM Tris-HCl (pH 7.4), 150 mM NaCl, 1 mM EDTA, 1 mM EGTA, 100 mM NaF, 150 mM sodium pyrophosphate, 2 mM sodium orthovanadate, 2 mM PMSF and protease inhibitor (Roche Diagnostics Corporation, Indianapolis, IN), using PRO 200 Laboratory Homogenizer (Pro Scientific). Homogenates were centrifuged at 12,000 RPM at 4°C for 20 min and supernatants were stored at -80°C until further analysis. Protein concentrations were determined using Bradford protein assay reagent according to the manufacturers' instructions (Bio-Rad Laboratories, Hercules, CA, USA). Forty μg of whole cell proteins were electrophoresed on a NuPage 4-12% gradient, Bis-Tris gel (Invitrogen, Carlsbad, CA) and were transferred on to nitrocellulose membranes. The membranes were blocked with Li-Cor blocking solution (Li-Cor Biosciences, Lincoln, NE), incubated overnight with either polyclonal rabbit anti-Iba1 (Wako Chemicals, Richmond, VA), anti-GFAP antibodies (Dako, Carpinteria, CA) or monoclonal mouse anti-β-actin (Sigma Chemicals, St. Louis, MO), washed and probed with secondary antibodies, IRDye 680LT conjugated Goat (polyclonal) anti-Mouse IgG (H+L) or IRDye 800CW conjugated Goat (polyclonal) anti-Rabbit IgG (H+L) (Li-Cor Biosciences). Membranes were kept moist and protected from light. Proteins were visualized and membranes were scanned using Odyssey Infrared Imaging System using Odyssey channel 800 for anti-Rabbit (green fluorescence) and channel 700 for anti-Mouse (red fluorescence) (LiCor Biosciences).

### Analysis of Sirt1 protein by western immunoblotting

Twenty μg of whole cell proteins were electrophoresed on 8% polyacrylamide gels and were transferred on to nitrocellulose membranes. The membranes were blocked with 5% milk, incubated overnight with polyclonal rabbit anti-Sirt antibody (Upstate, Millipore, Billerica, MA), washed and probed with a secondary donkey anti-rabbit IgG (Santa Cruz Biotechnology, Santa Cruz, CA) conjugated to horseradish peroxidase (Santa Cruz Biotechnology). Blots were stripped using ReBlot Plus mild stripping solution (Millipore, Billerica, MA) and probed for β-actin using monoclonal β-actin (Sigma Chemicals, MO) and goat anti-mouse IgG (Santa Cruz Biotechnology) antibodies. Proteins were visualized using commercially available electrochemiluminescence kit (Amersham Biosciences, Piscataway, NJ, USA). Protein bands were scanned using the Molecular Imager Gel Doc XR System (Bio-Rad) and intensities were analyzed using the Discovery Series Quantity One 1-D Analysis Software (Bio-Rad).

### Analysis of plasma antioxidant and inflammation markers

Plasma SOD, catalase, GPx and GSH levels were analyzed as mentioned above. Systemic inflammation was analyzed by measuring plasma cytokine levels using the RayBio Mouse Cytokine Antibody Array 3 (RayBiotech Inc., Norcross, GA). The assay employs a qualitative dot blot technique. The standard array matrix consisted of a 14 × 10 dot grid on a nitrocellulose membrane with 62 unique antibodies. Assay was conducted as per the manufacturers' protocol. Briefly, membranes were individually placed in chambers of eight-well tissue culture plates and blocked with the blocking buffer provided by the manufacturer. After blocking, membranes were incubated for 2 hr with 1:10 diluted plasma using blocking buffer supplied by the manufacturer. The membranes were then incubated with biotin-conjugated antibodies for 2 hr and horse radish peroxidase conjugated streptavidin for 1 hr. Proteins were visualized using SuperSignal West Pico enhanced chemiluminescent solution (Thermo Scientific, Rockford, IL, CA, USA). Protein bands were scanned using the Molecular Imager Gel Doc XR System (Bio-Rad) and intensities were analyzed using the Discovery Series Quantity One 1-D Analysis Software (Bio-Rad). Intensities of positive controls were used to normalize the results from different membranes. All membranes were processed and developed simultaneously to minimize variability.

### Chemical analysis to quantitate major polyphenols of BM extract

Phenolic constituents of BM extracts were determined by LC-MS analyses, using a triple quad and a time of flight analyzer. In brief, one gram of powder, derived from the freeze-dried BM juice, was extracted with 50 mL of methanol for 30 min using an ultrasound water bath. The mixture was filtered to remove the particulates, and the solvent was evaporated in a rotary evaporator at 30°C yielding 233 mg of dry extract. The residue was re-dissolved in 5 mL of LCMS grade methanol and filtered through a 0.22 micron syringe filter. The solution was transferred into a LCMS vial and 1 μL was injected into the LC-QQQ-MS instrument for quantitative analysis carried out using an 6400 QQQ-MSD instrument connected to an Agilent 1200 LC system (Agilent Technologies, Foster City, CA). Chromatographic analysis was performed on an Zorbax XDB C18 column (1.8 μm, 4.6 × 50 mm, Agilent Technologies) using a gradient of 10-100% acetonitrile containing 0.01% formic acid and water containing 5 mM ammonium formate with 0.01% formic acid over 15 minutes (flow rate 1 mL/min). The column was heated to 40°C. For quantitative analysis the QQQ instrument was operated in dynamic MRM mode using an ESI ion source with capillary voltage at 2500 V. The gas temperature was kept constant at 350°C with a gas flow of 12 L/min. Nebulizer gas pressure was set to 30 psi. Standard solutions (10 μg/mL) of caffeine, *trans*-chalcone, flavone, caffeic acid, catechin, quercetin and gallic acid were prepared in methanol and calibration curves were generated using injection volumes of 0.1, 0.2, 0.5, and 1 μL. Quantitation was conducted using the Masshunter Workstation Qualitative and Quantitative Analysis software version B.01.03 (Agilent Technologies). High resolution TOFMS data (1 μL injection) was recorded on a 6210 LC/MSD-TOF instrument equipped with an 1100 chromatography module (Agilent Technologies). The TOF instrument was operated with a capillary voltage of 4500 V, a gas temperature of 300-350°C with a flow rate of 10 L/min and a nebulizer pressure of 30 psi. Chromatographic conditions and data analysis were conducted as described above for quantitative analysis.

### Statistical analysis

Statistical analysis was conducted using GraphPad Prism, Prism 5 for Windows, version 5.01. All data were expressed as mean values ± SEM. A one-way ANOVA model was used to compare means between the three animal groups (control, HFD and HFD + BM). For each sample, duplicate determinations were conducted. *Post hoc *pair-wise multiple comparisons were evaluated using the Tukey's Multiple Comparison test, after ANOVA. Results were considered significant at p < 0·05.

## Results

### BM modulates metabolic parameters

We have previously demonstrated that BM has no significant effect on food or water intake, reduced overall weight gain by 21% as compared to mice fed HFD with a concomitant reduction in hepatic and plasma lipids and improvement in glucose tolerance [19]. The brain samples used in the current study were obtained from the same mice cited in our published studies [19]. Although epidemiological observations suggest an effect of obesity on brain volume reduction, our animal studies indicated no difference in the brain weights among the different treatment groups (data not shown).

### BM prevents HFD-induced leakage of Evans blue dye

**Figure 1:**
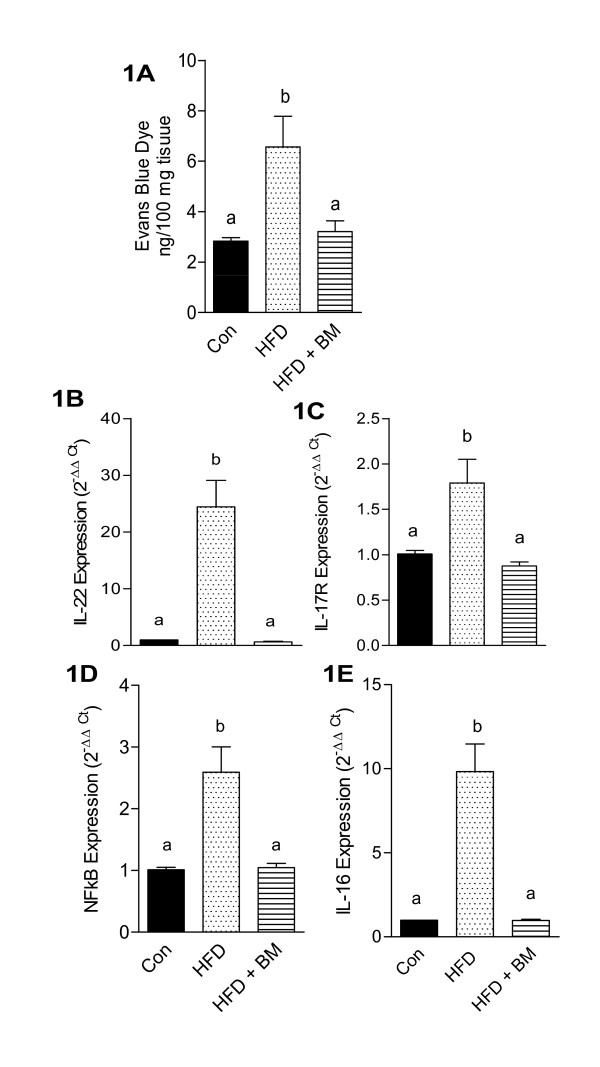
**Effect of HFD and BM on blood-brain barrier permeability and neuroinflammatory markers**. Concentrations of Evans blue dye (A) and mRNA expression of IL-22 (B), IL-17R (C), NF-κB1 (D) and IL-16 (E) in brains of mice fed HFD with and without BM. Values represent the mean ± SE (n = 4). Each sample was analyzed in duplicate, in two independent experiments. ^a,b,c^Mean values with common letters do not differ (p < 0·05).

Figure 1A demonstrates a significant increase (p < 0.05) in the amount of Evans blue dye in the brains of mice fed HFD for 16 weeks as compared to control. BM normalized the amount of Evans blue dye in brains of mice fed HFD with BM (Figure 1A).

### Microarray analysis of whole brain mRNA expression

Out of 39,000 transcripts, more than 4,000 genes were differentially regulated by HFD, out of which 1,100 genes were up-regulated and 1,200 genes were down-regulated by 2-fold or more (data not shown). An independent manuscript is being prepared depicting the global overview of results and categorizing significant pathways of metabolic and cellular functions. The primary focus of this manuscript is to investigate the effects of BM on pro-inflammatory cytokines and molecules regulating cellular stress pathways. Table 2 demonstrates fold-change of selected gene expression involved in stress, inflammation, and transcription/signaling, in the brains of mice fed either HFD or HFD and BM, as compared to mice fed control diet. Expressions of selected genes were validated by measuring qRT-PCR, enzyme activities or western immunoblotting. Preliminary studies with Sirt1 expression demonstrated that trends of mRNA expression and protein levels were similar. Since the quantity of brain tissue available for testing was limited, we analyzed the remainder of the molecules by measuring only mRNA expression.

**Table 2:** Changes in brain gene expression of C57BL/6 female mice fed HFD ± BM

Gene Name	Symbol	Con	HFD	HFD + BM
Sirtuin 1	Sirt1	1.0	0.86	0.88

Sirtuin 3	Sirt3	1.0	0.72	1.28

Sirtuin 7	Sirt7	1.0	0.99	1.14

Forkhead box O3a	Foxo3a	1.0	2.92	1.32

Forkhead box O1	Foxo1	1.0	0.65	0.5

Superoxide dismutase 2, mitochondrial	Sod2	1.0	1.32	1.48

Glutathione S-transferase, pi 1	Gstp1	1.0	0.76	1.03

Glutathione peroxidase 4	Gpx4	1.0	0.9	1.10

*Interleukin 10	Il10	1.0	1.76	Absent

*Interleukin 16	Il16	1.0	16.06	Absent

*Interleukin 22	Il22	1.0	10.80	Absent

*Interleukin 17 receptor B	Il17rb	1.0	0.597	Absent

*Nuclear factor of kappa B1	NfκB1	1.0	8.22	Absent

Eukaryotic translation initiation factor 2, subunit 2 (beta)	Eif2s2	1.0	0.77	1.03

Eukaryotic translation initiation factor 2B, subunit 2 beta	Eif2b2	1.0	0.90	1.04

Eukaryotic translation initiation factor 2, subunit 3, structural gene X-linked	Eifs3x	1.0	0.79	1.2

Eukaryotic translation initiation factor 3, subunit 10 (theta)	Eif3s10	1.0	0.75	1.09

DnaJ (Hsp40) homolog, subfamily B, member 1	Dnajb1	1.0	0.74	0.94

DnaJ (Hsp40) homolog, subfamily C, member 2	Dnajc2	1.0	0.60	0.81

DnaJ (Hsp40) homolog, subfamily A, member 3	Dnaja3	1.0	0.55	1.53

DnaJ (Hsp40) homolog, subfamily C, member 7	Dnajc7	1.0	0.77	0.99

Heat shock protein 110	Hsp110	1.0	0.61	1.13

Heat shock 70 kDa protein 14	Hsp14	1.0	0.57	0.9

*Absent in control

### BM protects brain from HFD-induced neuroinflammation

HFD-induced neuroinflammation is characterized by BBB disruption, transmigration of activated lymphocytes as well as activation of microglial cells by infiltrating immune cells leading to increased production of pro-inflammatory cytokines such as IL-16 [30,31]. In the present study, we observed a 10-fold up-regulation of IL-22 mRNA expression (Figure 1B, p < 0.05) along with a significant up-regulation of IL-17R expression by 177% (Figure 1C, p < 0.05) in the brains of mice fed HFD. However, the 10-fold increase in IL-22 mRNA expression detected by qRT-PCR was lower than the 16-fold increase observed by microarray analysis (Table 2). HFD also significantly up-regulated the mRNA expression of NF-κB1, a "master inflammatory switch" and activator of microglial cells, by 156% as compared to control mice, which was normalized in mice fed HFD with BM (Figure 1D, p < 0.05). Feeding HFD for 16 weeks, increased IL-16 mRNA expression by 10-fold as indicated by microarray analysis (Table 2) and 24-fold by qRT-PCR (Figure 1E, p < 0.05). Both, IL-22 and IL-16 were absent in the brains of control mice and those fed with HFD with BM (Figure 1B and 1E, respectively).

**Figure 2:**
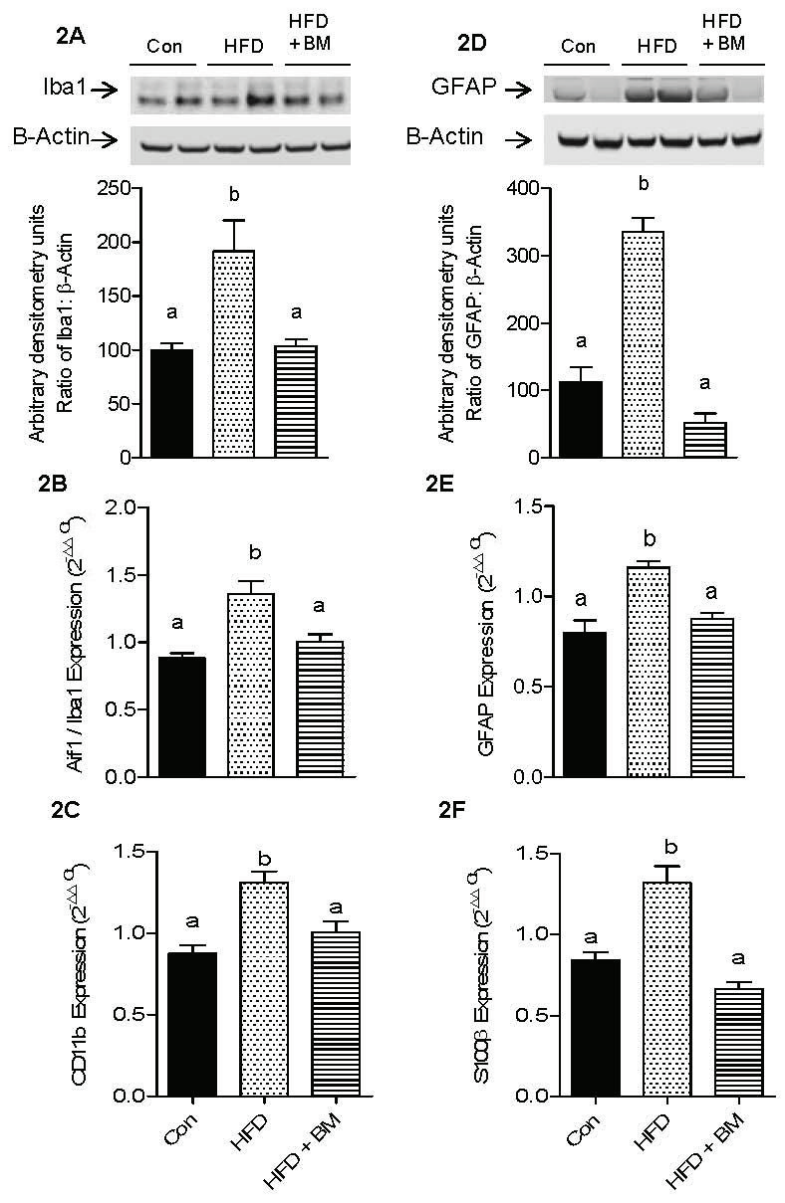
**Effect of HFD and BM on glial cell activation markers**. Figure 2 represents microglial activation markers, Iba1 protein levels (A), Aif1/Iba1 mRNA expression (B) and CD11b mRNA expression (C) as well as astrocyte activation markers, GFAP protein levels (D), GFAP mRNA expression (E) and S100β mRNA expression (F). The bar graph in Figure 2A and 2D represents the densitometry scan of 17-kD Iba1 and 50-kD GFAP proteins, respectively. Values for qRT-PCR represent the mean ± SE (n = 4), while those for western blot analysis represent the mean ± SE (n = 3). Each sample was analyzed in duplicate, in two independent experiments. ^a,b,c^Mean values with common letters do not differ (p < 0·05).

Activation of microglial cells by HFD was indicated by modest, but significant upregulation of Aif1/Iba1 and CD11b mRNA levels by 140-150% as compared to control, (Figures 2B and 2C, respectively, p < 0.05). Similarly, mRNA expression of GFAP and S100B were also up-regulated by 140-150% above that of control, indicative of astrocyte activation (Figures 2E and 2F, respectively, p < 0.05). Interestingly GFAP proteins were up-regulated by more than 300% above that of control in contrast to a modest 174% increase in Iba1 protein levels above that of control (Figures 2D and 2A, respectively, p < 0.05). Although the exact mechanisms remain unclear, BM inhibited markers of glial cells activation (Figure 2) and normalized the cytokine expression of IL-17R, IL-16, IL-22 and NF-κB1 in brains of mice fed HFD (Figure 1), suggesting an anti-neuroinflammatory effect in these mice.

### BM attenuates HFD-induced oxidative stress in brain

**Figure 3:**
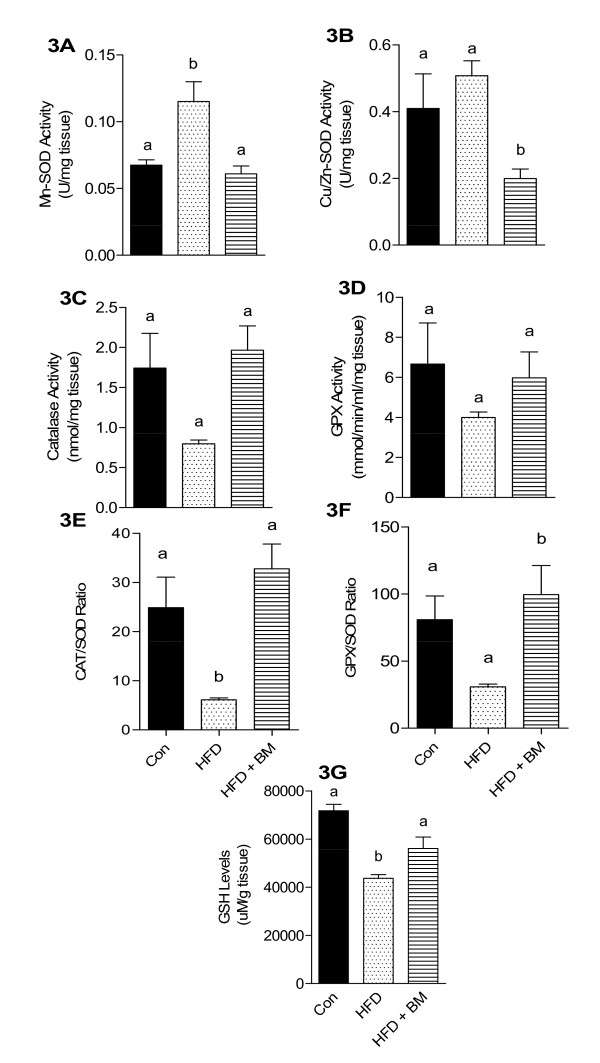
**Effect of HFD and BM on brain anti-oxidative enzymes**. Enzymatic activities of MnSOD (A), CuZnSOD (B), catalase (C), GPX (D), ratios of CAT/SOD (E) and GPx/SOD (F) as well as GSH (E) levels in brains of mice fed HFD with and without BM. Values represent the mean ± SE (n = 4). Each sample was analyzed in duplicate. ^a,b,c^Mean values with common letters do not differ (p < 0·05).

Figure 3 demonstrates the effect of BM on HFD-induced oxidative stress in whole brain as measured by antioxidant enzyme levels. HFD significantly induced mitochondrial MnSOD enzyme activity by 78% as compared to control (Figure 3A, p < 0.05), while 24% induction of CuZnSOD was not significant (Figure 3B, p > 0.05), as compared to control mice. BM normalized the MnSOD activity (Figure 3A), but significantly reduced CuZnSOD activity as compared to both, HFD-fed and control mice (Figure 3B, p < 0.05). Hydrogen peroxide generated by increased ROS production is detoxified by either catalases (CAT) or GPx. In our study, both catalase (Figure 3C) and GPx (Figure 3D) were reduced by 54% and 40%, respectively, in HFD-fed mice, both of which were normalized in mice fed HFD with BM. However, changes in catalase and GPx failed to achieve significance due to large sample variability. The CAT/MnSOD ratio was significantly reduced by 75% in the brains of HFD-fed mice compared to control, while simultaneous feeding of BM increased the CAT/MnSOD ratio by 131% as compared to control and by more than 500% as compared to HFD-fed mice (Figure-3E, p < 0.05). In contrast, HFD non-significantly reduced GPX/MnSOD ratios by 68% (Figure 3F). BM normalized the brain GPX/MnSOD ratios as compared to control mice, but significantly up-regulated the ratios by 323% compared to HFD-fed mice (Figure 3F, p < 0.05). Furthermore, the ratios of CAT/total SOD (sum of MnSOD and CuSOD) were insignificantly reduced in brain of HFD-fed mice as compared to control (data not shown) but significantly reduced by 80% compared to mice fed HFD with BM (data not shown). Overall, ratios of GPX/total SOD were non-significantly reduced in brains of HFD-fed mice compared to control and those fed HFD with BM (data not shown). BM also up-regulated HFD-associated reduction in brain GSH levels (Figure 3G).

**Figure 4:**
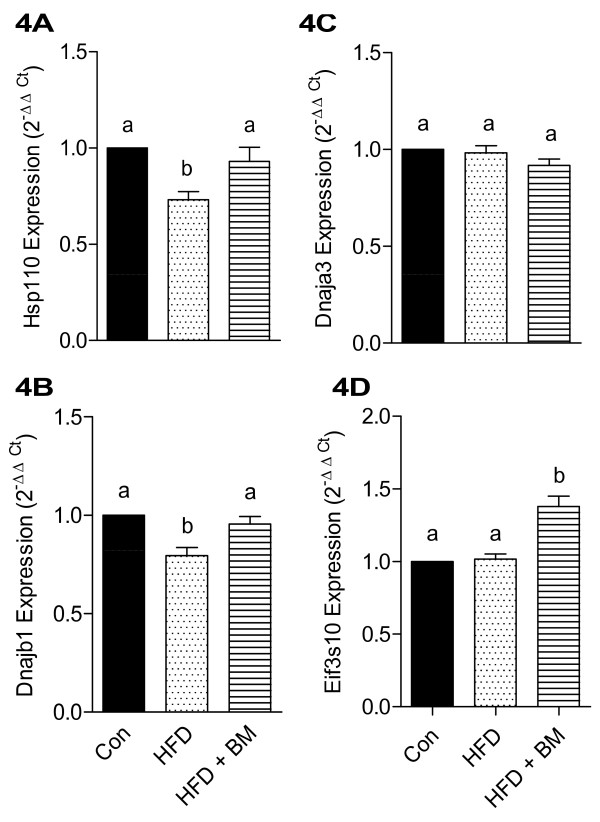
**Effect of HFD and BM on brain chaperone molecules**. mRNA expression of brain chaperone molecules, Hsp110 (A), Dnajb1 (B), Dnaja3 (C), and Eif3s10 (D) in mice fed HFD with and without BM. Values represent the mean ± SE (n = 4). Each sample was analyzed in duplicate in two independent experiments. ^a,b,c^Mean values with common letters do not differ (p < 0·05).

We also observed a significant reduction in stress proteins, such as heat shock protein 110 (Hsp110) and Dnajb1 (Hsp40) after feeding HFD for 16 weeks. Figure 4 demonstrates the effects of HFD and BM on Hsp110 (Figure 4A), Dnajb1 (Figure 4B), Dnajab3 (Figure 4C) as well as Eukaryotic translation initiation factor (Eif3s10) (Figure 4D). HFD significantly reduced mRNA expression of HSp110 and Dnajb1 by 27% and 21%, respectively, as compared to control (Figures 4A and 4B, p < 0.05), while BM normalized expression of both genes. Both, HFD and BM had no significant effect on the expression of Dnajab3 and eif3s10 (Figures 4C and 4D, respectively).

### BM differentially regulates Sirt and FoxO expression in brains of HFD-fed mice

**Figure 5:**
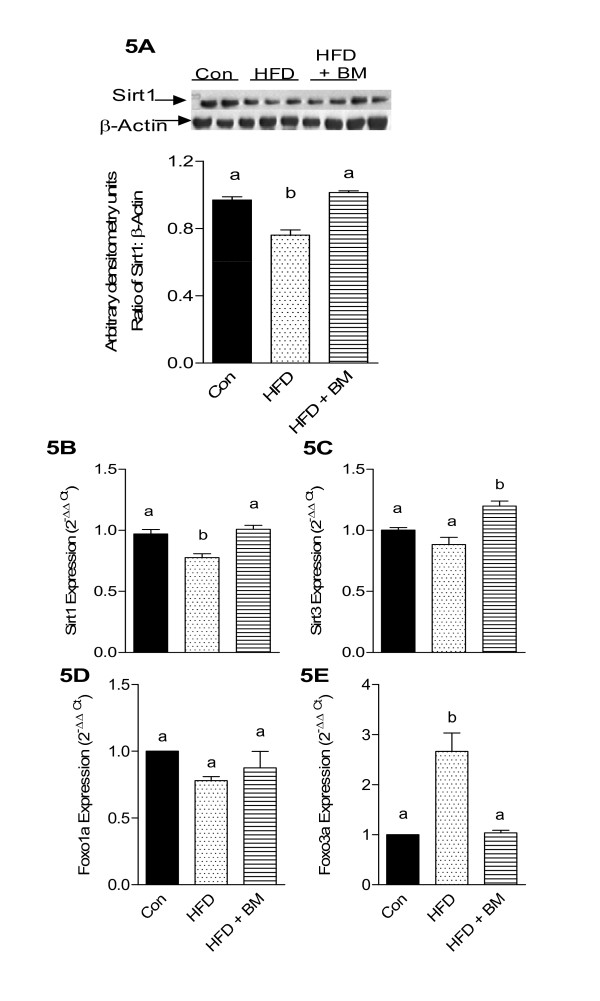
**Effect of HFD and BM on brain Sirt and FoxO protein expression**. Whole brain Sirt1 levels in mice fed HFD with and without BM. Figure 5A represents western blot of Sirt1 protein levels and Figure 5B demonstrates mRNA expression. The bar graph in Figure 5A represents the densitometry scan of 110-kD protein (n = 4). Figure 5C demonstrates Sirt3 mRNA expression analyzed by qRT-PCR (n = 4), while FoxO1 and FoxO3a mRNA expression are depicted in Figures 5D and 5E, respectively (n = 4). Values represent the mean ± SE. Each sample was analyzed in duplicate. ^a,b,c^Mean values with common letters do not differ (p < 0·05).

Feeding HFD has been demonstrated to reduce hippocampal levels of stress-resistant gene, Sirt1, involved in regulating oxidative stress, as well as improving neuronal insults and synaptic plasticity in the brain. Since our results indicated that BM reduces neuroinflammatory markers as well as oxidative stress, we further tested the hypothesis that BM will regulate anti-stress genes such as Sirt and FoxO in HFD-fed mice. It was observed that HFD significantly reduced Sirt1 mRNA expression levels by 20% (p < 0.05) as compared to control mice (Figure 5B). To investigate the correlation between mRNA expression and protein levels, Sirt 1 protein expression was analyzed by western immunoblotting. A comparable trend was also observed wherein the brain Sirt protein levels were reduced by 25% (p < 0.05) (Figure 5A) in HFD-fed mice as compared to control mice. Feeding of BM normalized both, Sirt1 mRNA expression as well as protein levels (Figures 5B and 5A, respectively). Similarly, the mRNA expression of mitochondrial Sirt isoform, Sirt3, was non-significantly reduced by 11% in HFD-fed mice as compared to control mice (Figure 5C). Changes in Sirt3 mRNA expression by qRT-PCR were similar to those observed by microarray analysis (Table 2). Interestingly, BM not only normalized the HFD-associated reduction of Sirt3 mRNA expression but also up-regulated it by 20% (p < 0.05) above that of control mice (Figure 5C).

Besides Sirt, resistance to oxidative stress is also governed by FoxO proteins. In our study, HFD had no significant effect on FoxO1 mRNA expression (Figure 5D). However, we observed a significant increase in FoxO3a expression by more than 2.5-fold (Figure 5E, p < 0.05) as compared to control, which was normalized in mice fed HFD with BM.

### BM normalized plasma antioxidants and reduced systemic inflammation

**Figure 6:**
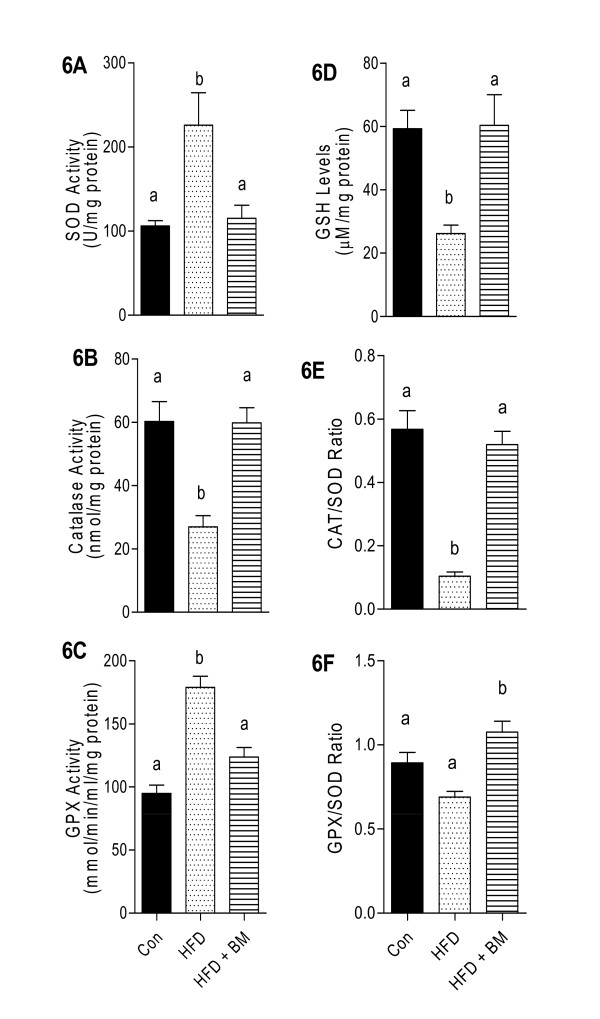
**Effect of HFD and BM on plasma anti-oxidative enzymes**. Enzymatic activities of SOD (A), catalase (B), GSH (D), and levels and ratios of GPX (C), CAT/SOD (E) and GPx/SOD (F) in plasma of mice fed HFD with and without BM. Values represent the mean ± SE (n = 4). Each sample was analyzed in duplicate. ^a,b,c^Mean values with common letters do not differ (p < 0·05).

Overall, the effect of BM and HFD on plasma oxidative stress biomarkers demonstrated a pattern similar to that observed in the brain. Figure 6A, indicated a 250% increase of plasma SOD levels in HFD-fed mice compared to control and 220% increase compared to mice fed HFD with BM (p < 0.05). Both, plasma catalase and GSH were significantly reduced in HFD-fed mice, while GPX was significantly increased as compared to control mice (Figures 6B, C and 6D, respectively). Feeding HFD with BM normalized plasma catalase, GPX and GSH to control levels (Figure 6A-6D). Similar to brain, plasma CAT/SOD ratios were significantly reduced by 40-60% with HFD feeding as compared to control and HFD + BM fed mice, respectively (Figure 6E, p < 0.05). Plasma GPX/SOD remained unchanged in all groups (Figure 6F).

**Figure 7:**
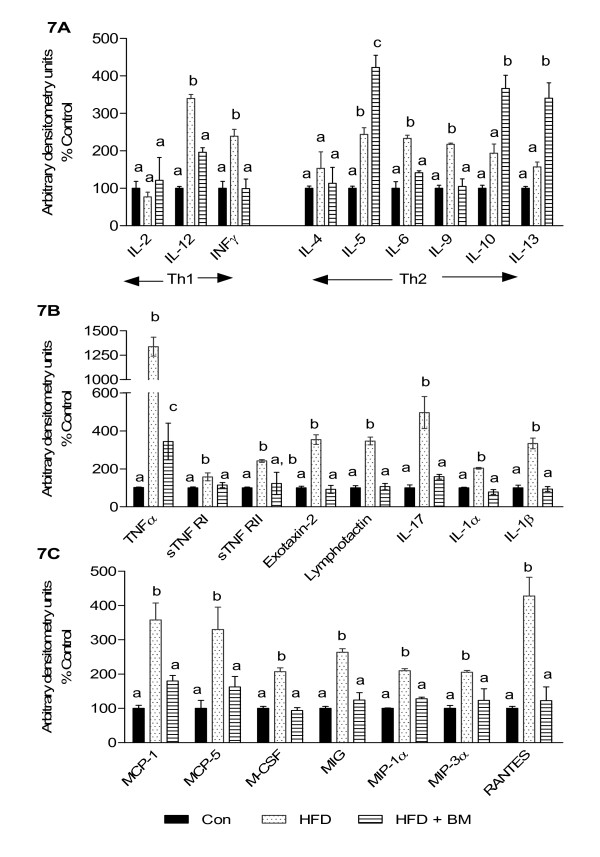
**Effect of HFD and BM on plasma inflammatory markers**. Effect of HFD and BM on plasma Th1 and Th2 cytokine levels (A), and other circulating inflammatory cytokines and chemokines (B and C). Values represent the mean ± SE (n = 4). Each sample was analyzed in duplicate. ^a,b,c^Mean values with common letters do not differ (p < 0·05).

Low-grade systemic inflammation plays an important role in the development of metabolic abnormalities including obesity, diabetes and cardiovascular diseases and may impact BBB integrity in HFD-fed mice [2,31-33]. We therefore investigated the effects of HFD and BM on peripheral inflammatory markers using a cytokine antibody array. As expected, HFD was observed to significantly increase IL-12 and interferon γ (IFNγ) levels with a concomitant increase in IL-6 and IL-9 levels as compared to the control mice (Figure 7A, p < 0.05). These pro-inflammatory cytokines were normalized in mice fed HFD with BM (Figure 7A). Although HFD was also seen to significantly increase IL-5, IL-10 and IL-13, these anti- inflammatory cytokines were further increased by feeding BM above that of control and HFD-fed mice (Figure 7A, p < 0.05). Additional inflammatory molecules such as tumor necrosis factor α (TNFα), soluble TNF receptor II (sTNFRII), exotaxin-2, lymphotactin, IL-17 and IL-1β were increased by157 to 1000% (Figure 7B, p < 0.05), along with 200 to 400% increases in monocyte chemoattractant protein 1 (MCP1), MCP-5, macrophage colony-stimulating factor (MCSF), macrophage inflammatory protein-1α (MIP-1α) and regulated on activation, normal T expressed and secreted (RANTES), member of IL-8 family of cytokines (Figure 7C, p < 0.05) in HFD-fed mice as compared to control animals. Mice fed HFD with BM demonstrated significantly lower cytokine levels as compared to HFD-fed mice (Figures 7B and 7C, p < 0.05).

### Phenolic compounds of BM

**Figure 8:**
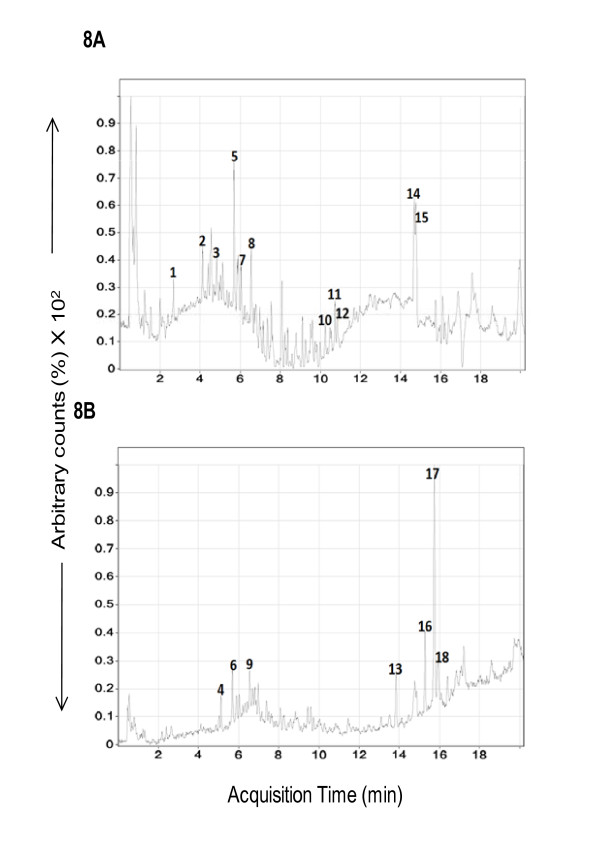
**LC-MS analysis of phenolic constituents in BM extract**. Figure 8 demonstrates ESI positive (8A) and negative (8B) total ion chromatograms of a representative BM extract.

The quantity and diversity of the phenolic constituents in the BM extracts were analyzed to provide a chemical fingerprint for future comparison. Qualitative analysis was initially undertaken to obtain a profile of the extract using TOF-MS to obtain high-resolution mass spectrometric data. The sample was analyzed in both positive and negative modes (Figures 8A and 8B, respectively) to identify marker ions, necessary for subsequent standardization. Seventeen prominent ions in the chromatogram were identified (Table 3) that can be used for comparative purposes in future studies. Compounds previously identified in the BM extract were quantitated using MRM on a QQQ-MS (Table 4). Individual compounds were identified by analysis of the LCMS chromatograms based on comparison with the retention times, ionization patterns, and fragmentation patterns of standards. Of the standards analyzed, catechin was the most abundant polyphenol (3%, w/w of the BM methanolic extract dry weight) followed by quercetin (0.62%, w/w), trans-chalone and caffeine (0.3 and 0.25%, w/w, respectively) (Table 4). Caffeic acid, gallic acid and flavones were identified at less than 0.01%, w/w in BM methanolic extracts (Table 4). Future studies are targeted to identify the undetermined peaks and the exact structure-activity relation of these fractions.

**Table 3:** Major Ions observed during Time-of-Flight analyses based on Figures 8A and 8B

Peak #	Retention Time (min)	*m/z*	Polarity	Species
1	2.7	394.35	Positive	Not Determined

2	4.1	340.261	Positive	[M+H]^+^

3	4.8	566.31	Positive	[M+H]^+^

4	5.1	966.0	Negative	Not Determined

5	5.7	419.33	Positive	[M+H]^+^

6	5.7	909.45	Negative	Not Determined

7	6.0	419.33	Positive	[M+H]^+^

8	6.0	327.22	Positive	[M+H]^+^

9	6.5	325.20	Negative	[M-H]^-^

10	10.2	496.34	Positive	Not Determined

11	10.7	4317.34	Positive	Not Determined

12	10.8	293.21	Positive	[M+H]^+^

13	13.8	227.23	Negative	Not Determined

14	14.7	282.28	Positive	[M+H]^+^

15	14.8	331.29	Positive	[M+H]^+^

16	15.3	367.27	Negative	Not Determined

17	15.7	255.23	Negative	Not Determined

18	15.9	281.25	Negative	Not Determined

**Table 4:** Quantitative analysis of a methanolic BM extract using LC-QQQ-MS

Abundance % (w/w)	Abundance % (w/w)	MRM Transition (Precursor or Product)	Polarity	Fragmentor	Collision Energy	Retention Time
**trans-Chalcone**	0.30	209/103	positive	130	40	10.2

**Flavone **	< 0.01	223/129	positive	130	40	8.4

**Caffeine **	0.25	195/123	positive	130	40	2.2

**Gallic acid **	< 0.01	169/125	negative	90	15	0.9

**Quercetin**	0.62	301/151	negative	90	15	5.5

**Caffeic acid **	< 0.01	179/135	negative	100	20	2.5

**Catechin **	3.02	289/245	negative	100	20	2.2

## Discussion

Considerable attention has been focused on the effects of HFD on activation of hypothalamic inflammatory responses [13,34-37]. However, earlier efforts were directed mostly towards the role of neuroinflammation on leptin and insulin resistance in the hypothalamus that may disrupt satietogenic and adipogenic responses to maintain a healthy body mass [38]. More recent advances suggest a role for HFD-induced neuroinflammation in impaired cognition and memory as well as neurodegenerative diseases such as dementia and Alzheimer's disease [39,40]. Although systemic or peripheral stress and inflammation may play a role in disrupting BBB, the brain, specifically the glial cells are capable of initiating an innate and local immune response including release of cytokines and ROS species that might be detrimental to neuronal health and ultimately reduce synaptic plasticity leading to cognitive impairment [41-43]. HFD-associated neuroinflammation is a complex process involving a multitude of pro- and anti-inflammatory molecules thereby requiring novel therapeutic approaches that will ameliorate not only obesity-associated metabolic perturbations, but also neuroinflammation.

### BM protects brain from HFD-induced neuroinflammation and glial cells activation

Glial cells, including astrocytes and microglia are key mediators of neuroinflammation. Hypothalamic inflammation in response to dietary insults such as saturated fatty acids is a result of activated microglial cells that increase the release of pro-inflammatory cytokines such as IL-16 [30,31,44]. Increased levels of Iba1 and GFAP indicate activation of resident microglial cells and astrocytes, respectively, possibly leading to the increased levels of IL-16 and IL-22 mRNA expression observed in the brains of HFD-fed mice. Neuroprotective effects of BM is indicated by reduced glial cells activation as well as normalized IL-16 and IL-22 mRNA levels in brains of mice fed HFD with BM. Similarly, neuroprotective effects of BM were also evident from a recent study which demonstrated that BM attenuated global cerebral ischemia-associated oxidative stress and normalized short-term memory and motor function in diabetic mice [45].

### BM regulates peripheral and neuroinflammatory cross-talk by modulating BBB

Increased expression of IL-22 in the brains of HFD-fed mice may indicate not only glial cells activation, but also the presence of activated Th17 cells since IL-22 can be secreted by active CD4+ Th17 cells [46-50]. Although brain is considered as an "immune privileged" organ, studies now indicate that brain can demonstrate an innate immune response to systemic infections and cerebral injury since activated Th17 cells can transmigrate through an intact BBB [51]. Alternatively, peripheral or systemic inflammation can contribute to maintaining glial cells activation by modulating BBB [2,31-33]. Recent studies have demonstrated that obesity can selectively modulate and promote the expansion of specific lineage of Th17 cells resulting in systemic inflammation [52]. The major cytokines that arise from T-helper cell (Th1) and Th17 CD4(+) T-cell differentiation consist of IFNγ as well as IL-17 and IL-22. BM reduced systemic inflammation by reducing secretion of plasma pro-inflammatory cytokines, INFγ and IL-17 along with TNFα, exotaxin-2, lymphotactin and IL-1β in mice fed HFD with BM. Interestingly, BM upregulated specific Th2 cytokines IL-5, IL-10 and IL-13, which have been demonstrated to protect obesity-associated inflammation [53]. In contrast, Th2 cytokines such as IL-4 is known to exacerbate HFD-associated hypothalamic inflammation and weight gain [2]. However, we did not observe any significant differences in IL-4 secretion in mice fed HFD with or without BM.

Although it is unclear as how exactly the activated T cells migrate across the BBB, systemic inflammatory molecules such as MCP1 has been shown to disrupt BBB integrity [22], while MIP-1α may promote transendothelial migration of T cells [23] and M-CSF is indicative of glial cells activation [54]. In our studies, increased levels of plasma MCP-1, MIP-1α and M-CSF may indicate a potential threat to BBB integrity during overnutrition. A compromised BBB permeability was evident by the presence of increased Evans blue dye in the brains of mice fed HFD, which was significantly lower in mice fed HFD with BM, possibly due to reduction in plasma levels of MCP-1, MIP-1α and M-CSF.

It is however, interesting to note that BBB does not necessarily inhibit lymphocyte entry, but actively restrict the entry of resting T cells and allow transmigration of only recently activated T cells ([55] and references within). Besides IL-22, secretion of IL-17 by activated Th17 cells can induce IL-17 receptors (IL-17R) on the BBB endothelial cells and alter BBB permeability [48] and promote transmigration of activated Th17 cells into the brain [49,50]. We observed that HFD also induced the expression of IL-17 cognate receptor, IL-17RB. Studies have demonstrated that IL-17 can promote neuronal injury through IL-17-IL-17R interactions [56]. It is important to note that since whole brains were dissected after perfusion of the mice with cold PBS, release of inflammatory cytokines through the blood into the brain is expected to be negligible. Reduced Evans blue dye in the brain and normalization of IL-22 and IL17R mRNA expression in the brains of mice fed HFD with BM strongly suggests that BM restores BBB permeability as well as prevents transmigration of Th17 cells. However, modulation of BBB permeability, thereby leading to immune cell trafficking by BM, requires further investigation.

### Antioxidative potential of BM

Zhang et al. offer important insights into the link between overnutrition and activation of inflammatory and stress pathways in the hypothalamus [36]. Hypothalamic neurons generally enriched with inactive IKKβ/NF-κB, become markedly activated due to overnutrition and consequently generate hypothalamic stress [36] and produce free radicals by activated microglial cells [57]. Similar to studies of Zhang et al. [36], our data indicates that HFD reduced the expression of stress proteins (Hsp110 and Dnajb1), with a concomitant increase in NF-κB1 mRNA expression as compared to control mice. HFD-induced oxidative stress and overproduction of reactive oxygen species (ROS) can lead to synaptic dysfunction and cognitive decline in mice [34,36,58]. Increased SOD activities observed in HFD-fed mice are indicative of adaptive mechanisms in response to increased levels of ROS [29], generating H_2_O_2 _that can be further detoxified by either catalases or GPx. In our study, HFD-fed mice were unable to detoxify increased production of H_2_O_2 _as evident by significant reduction in both, catalase and GPx enzyme activities. Endogenous GSH also plays an important role in detoxifying ROS. Lower GSH levels in brains of HFD-fed mice is indicative of ROS-induced tissue damage which could be possibly due to reduced capacity to cope with oxidative damage as reflected by a lower CAT/SOD and GPX/SOD ratios. A similar trend in systemic oxidative stress markers is observed in mice fed HFD, while BM was noted to reduce plasma SOD and GPX and normalize plasma GSH, catalase as well as CTA/SOD and GPX/SOD ratios.

Our results are similar to those demonstrated with water extracts of BM fruits that prevented acetaminophen-, furosemide- and chloroform-induced hepatotoxicity in mice [59], by attenuating the production of lipid peroxidation product, malondialdehyde (MDA) and increasing hepatic glutathione (GSH), indicative of its antioxidant properties [59]. BM seed extracts also improve antioxidant profile in streptozotocin (STZ)-induced diabetic male Wistar rats by modulating GSH, catalase, GPx and glutathione-s-transferase enzyme activities [60]. BM was observed to normalize both, anti-oxidative enzymes as well as stress proteins in brains of mice fed HFD with BM. Brain Hsp are involved in synaptic plasticity and may be involved in protecting brain either against stress-induced apoptosis [61] or unfolded protein response (UPR) [62]. In our study, increase in Hsp70 and Dnajb1 proteins by BM may suggest attenuation of either neuronal stress or UPR, induced by HFD.

### Modulation of Sirt and FoxO expression by BM

NF-κB, a "master inflammatory switch" is "turned on" by HFD [36] and "turned off" by the "master metabolic switch", Sirtuin 1 (Sirt1) [63]. Modulation of neurodegenerative diseases by Sirt1 is associated with improvement of oxidative stress and neuronal inflammation [64]. Feeding of HFD to Sprague-Dawley rats reduced Sirt1 levels in hippocampus and cerebral cortex due to increased oxidative stress [12]. The observed 25% reduction of Sirt1 protein in brains of HFD-fed mice is lower than the 81% reduction reported by Wu et al. [12] in the hippocampus and cerebral cortex of HFD-fed rats. The observed differences in the levels of Sirt inhibition in our study and that of Wu et al. [12] is possibly due to the fact that regulation of Sirt1 expression differs in various brain regions and is mostly up-regulated in the hippocampus and cerebral cortex [65]. While Wu et al. [12] analyzed the effects of HFD in the hippocampus and cerebral cortex, our study was conducted using an aliquot of the whole brain rather than hippocampus or cerebral cortex. While, HFD had no effect on Sirt3, BM induced a minimal but significant increase in Sirt3 mRNA expression. Sirt3 is a mitochondrial deacetylase and the murine Sirt3 transcript codes for three protein variants [66]. The exact role of BM-induced Sirt3 mRNA expression, as well as the protein variants requires further investigation.

Sirt is a critical regulator of FoxO proteins in response to oxidative stress [67,68], which are now emerging as an important target in mechanisms of neurodegenerative diseases. Increased levels of neuronal FoxO3a have been associated with neuronal cell death [69] as well as oxidative stress [70]. In our study, HFD-induced increase in FoxO3a may have contributed to either increased oxidative stress or to microglial cells activation via NF-κB. It is possible that concomitant increase in Sirt1 and reduction in FoxO3a may have lowered oxidative stress and NF-κB expression in mice fed HFD with BM [12].

### Immunomodulatory potential of BM

Immunomodulatory potential of BM was evident by differential secretion of Th1, Th2 and Th17-associated pro-and anti-inflammatory cytokines in mice fed HFD with BM. In our studies, increased secretion of anti-inflammatory cytokine, IL-10 by BM is similar to that observed by Manabe et al who also demonstrated an increased secretion of IL-10, and transforming growth factor β (TGF-β) in healthy rats fed BM for three weeks [71] along with *in vitro *increase of IL-10 secretion in human monocytes, THP-1 [71]. Similarly, the inhibitory effects of BM on IL-6, IL-1β and TNF-α observed in our study was also demonstrated in previous *in vitro *studies, wherein BM increased IL-10 production and inhibited IL-1β, IL-6 and tumor necrosis factor alpha (TNF-α) levels in lipopolysaccharide (LPS)-stimulated murine peritoneal macrophages, suggesting a prophylactic effect on LPS-induced inflammation [21].

### Chemical components of BM

BM is a mixture of various flavanoids, bitter and non-bitter cucurbitane, triterpene aglycones and/or glycosides, imparting antioxidant properties. Our studies demonstrate the presence of marker polyphenols such as catechin, quercetin, trans-chalone and caffeine, while Caffeic acid, gallic acid and flavones were present at lower concentrations. Similarly, Horax et al have also demonstrated catechin as a major polyphenol in pericarp and seeds of BM [72].

To our knowledge our study is the first to demonstrate a neuroprotective effect of BM in mice fed HFD, which demonstrated significant reduction in BBB leakage, neuroinflammatory cytokines mRNA expression (IL-16, IL-22), glial cells activation and NF-κB1 expression, as well as improved oxidative stress in the brains of mice fed HFD with BM, along with amelioration of peripheral oxidative stress and systemic inflammation.

Overall, induction of oxidative stress, modulation of longevity and ER-chaperone genes, as well as induction of interleukins and NF-κB1 by HFD supports the role of obesity in the pathogenesis of neuroinflammation. Our study also presents novel properties of BM that offer unique treatment options to ameliorate not only obesity, hyperglycemia and hyperlipidemia, but also to prevent HFD-induced neuroinflammation associated with microglial cells activation and lymphocyte infiltration. This study also demonstrates ameliorating effects of BM on systemic inflammation and modulation of Th1, Th2 and Th17 immunity. Over the long run, studies such as ours can help to develop effective nutritional strategies to update and/or modify dietary recommendations and nutritional policies for "healthy aging". Future studies are warranted to identify the neuro-immunomodulatory and anti-inflammatory components of BM.

## Conclusions

As expected, HFD-fed mice demonstrated a reduction in Sirt1 protein levels with an increase in oxidative-stress and a simultaneous induction of FoxO3a, NF-κB1 and IL-16 mRNA expression. HFD was further observed to activate resident glial cells and increase proinflammatory cytokines, IL-16 and IL-22 possibly via NF-κB mediated mechanisms. BM significantly increased Sirt3 mRNA expression and normalized Sirt1 protein levels as well as oxidative stress in mice fed HFD with BM, suggesting antioxidant effect of BM. In addition, reduction and normalization of Iba1, GFAP, NF-κB1, IL-16 and IL-22 mRNA expression by BM, further suggests that BM inhibits microglial cells activation possibly by Sirt-NF-κB interactions. Our studies also demonstrate that BM can prevent not only HFD-induced neuroinflammation characterized by BBB disruption, lymphocyte transmigration across BBB and microglial cells activation, but also systemic stress and inflammation. Strategies to prevent not only weight gain, but also obesity-associated brain dysfunction are limited. BM offers a novel adjunct therapeutic approach to ameliorate obesity-associated peripheral inflammation and neuroinflammation.

## Competing interests

The authors declare that they have no competing interests.

## Authors' contributions

PVN conceived and designed the study as well as analyzed and interpreted the data, and wrote the manuscript. LMJ, LB, GK, EV, RS, PS, and DF performed experiments for data acquisition. PW was involved in writing methods and results for chemical analysis of BM as well as LC-MS data analysis. VRN was involved in overall data analysis and critically revising the manuscript for important intellectual content. PVN has primary responsibility for final content. The authors have read and approved the final version of this manuscript.

Partial data was presented as a poster at the April 2009 ASBMB Annual meeting, as an oral talk at the April 2009 College of Tropical Agriculture and Human Resources' (CTAHR) annual symposium, Honolulu, HI and data on neuroinflammatory markers was presented at the Society on NeuroImmnue Pharmacology, 17^th ^Scientific Conference, April 2011, Clearwater Beach, FL.

## Abbreviations

AD: Alzheimer's disease; BBB: blood-brain barrier; BM: bitter melon; CD11b: Integrin αM; Cu/ZnSOD: copper/zinc SOD; FoxO: forkhead box class O transcription factor; GFAP: glial fibrillary acidic protein; GPx: glutathione peroxidase; GSH: glutathione; HFD: high-fat diet; Iba1: ionized calcium-binding adapter molecule 1; IL: interleukin; IL-17R: IL-17 receptor; MCP: monocyte chemoattractant protein 1; M-CSF: macrophage colony-stimulating factor; MIP-1α: macrophage inflammatory protein-1α; MnSOD: mitochondrial S100β: calcium binding protein, beta; RANTES: Regulated on Activation, Normal T Expressed and Secreted; SOD; PBS: phosphate-buffered saline; qRT-PCR: quantitative real-time reverse transcriptase-PCR; Sirt1: sirtuin 1; SOD: superoxide dismutase; T2D: type 2 diabetes; TGF-β: transforming growth factor β
